# Hybrid device of hexagonal boron nitride nanoflakes with defect centres and a nano-fibre Bragg cavity

**DOI:** 10.1038/s41598-021-03703-z

**Published:** 2022-01-07

**Authors:** Toshiyuki Tashima, Hideaki Takashima, Andreas W. Schell, Toan Trong Tran, Igor Aharonovich, Shigeki Takeuchi

**Affiliations:** 1 Department of Electronic Science and Engineering, Kyoto University, Kyoto, 615-8510 Japan; 2 Faculty of Mathematics and Physics, Leibniz University Hannover, 30167 Hannover, Germany; 3 Physikalisch-Technische Bundesanstalt, 38116 Braunschweig, Germany; 4 School of Mathematical and Physical Sciences, University of Technology Sydney, Ultimo, New South Wales 2007 Australia; 5 ARC Centre of Excellence for Transformative Meta-Optical Systems (TMOS), University of Technology Sydney, Ultimo, New South Wales 2007 Australia

**Keywords:** Nanoscience and technology, Optics and photonics

## Abstract

Solid-state quantum emitters coupled with a single mode fibre are of interest for photonic and quantum applications. In this context, nanofibre Bragg cavities (NFBCs), which are microcavities fabricated in an optical nanofibre, are promising devices because they can efficiently couple photons emitted from the quantum emitters to the single mode fibre. Recently, we have realized a hybrid device of an NFBC and a single colloidal CdSe/ZnS quantum dot. However, colloidal quantum dots exhibit inherent photo-bleaching. Thus, it is desired to couple an NFBC with hexagonal boron nitride (hBN) as stable quantum emitters. In this work, we realize a hybrid system of an NFBC and ensemble defect centres in hBN nanoflakes. In this experiment, we fabricate NFBCs with a quality factor of 807 and a resonant wavelength at around 573 nm, which matches well with the fluorescent wavelength of the hBN, using helium-focused ion beam (FIB) system. We also develop a manipulation system to place hBN nanoflakes on a cavity region of the NFBCs and realize a hybrid device with an NFBC. By exciting the nanoflakes via an objective lens and collecting the fluorescence through the NFBC, we observe a sharp emission peak at the resonant wavelength of the NFBC.

## Introduction

Photonic quantum technologies, such as photonic quantum computing and quantum sensing, have attracted considerable attention recently. In realizing these technologies, it is important to couple photons to single-mode optical fibres without connection loss. In this context, solid-state quantum emitters coupled with single mode fibres are of interest. Especially, optical nanofibres^1^, which are tapered fibres with a diameter less than the wavelength of the propagation light at the tapered waist, are promising. A nanofibre has high transmittance of almost unity, lossless interconnection to single mode fibres, and a large evanescent field in the tapered waist, and can efficiently couple photons emitted from quantum emitters on the surface to a single mode fibre with a coupling efficiency of about 30%^2,3^. The coupling of various kinds of solid-state quantum emitters with optical nanofibres has been demonstrated^2,4–8^.

Although the coupling efficiency of 30% is high, further improvement of the coupling efficiency toward 100% is desirable. For this purpose, nanofibre Bragg cavities (NFBCs), which are optical microcavities embedded in a nanofibre, have recently been proposed and demonstrated^9–14^. NFBCs have a single resonant mode with a small mode volume (on the order of the cube of the wavelength), high quality (Q) factors, ultra-wide tunability of the resonant wavelength, and lossless coupling to single-mode fibres^11^. Furthermore, we demonstrated the enhancement of photon emission from a single colloidal CdSe/ZnS quantum dot coupled with an NFBC^11^. However, the inherent photo-bleaching and blue-shift of the emission wavelength of colloidal quantum dots are problems. Moreover, the linewidth of the emission peak is typically more than 10 nm at room temperature, which is too broad for the resonant linewidth of an NFBC with a reasonable Q factor. Therefore, it is desired to couple stable and robust quantum emitters with a narrow emission peak to NFBCs.

For these quantum emitters, a thin layered material composed of hexagonal boron nitride (hBN) with defect centres has attracted attention. These emitters exhibit ultra-bright single photon emission with a quantum efficiency of over 90%^15^ and high robustness^16^. The defect centres in hBN show sharp emission peaks with line widths less than 5 nm at room temperature^17–20^ and a few nm at cryogenic temperature^21–24^. In addition to basic optical characteristics investigations, two-photon absorption using near-infrared lasers^25^ and anti-Stokes excitation^26^ has been realized. Also, a three-dimensional analysis of the dipole orientation of a single defect centre in hBN has been experimentally performed^15,27^. Moreover, the defect centres in hBN have been coupled to nanophotonic devices, such as optical nanofibres, waveguides, metamaterials, and photonic crystals^28–32^.

In this paper, we report on the experimental realization of a hybrid device of NFBCs and ensemble defect centres in hBN nanoflakes. In this experiment, we fabricate NFBCs with a Q factor of 807 and a resonant wavelength at around 573 nm, which almost matches the fluorescent wavelength of the defect centres, by helium-focused ion beam (FIB) system. Using the helium FIB, the problem of residual ions in the device, exhibited by a previous device created using a gallium FIB, has been solved, resulting in better resonator characteristics^33^. A hybrid device with an NFBC and defect centres in hBN nanoflakes is then realized by picking and placing the hBN nanoflakes on a cavity region of the NFBC using a custom-made manipulation system. Finally, we confirm a sharp emission peak at the resonant wavelength of the NFBC when we excite the nanoflakes in the hybrid device via an objective lens and detect the photons through the fibre output of the device.

## Results and discussion

### Optical properties of defects in hBN nanoflakes

**Figure 1:**
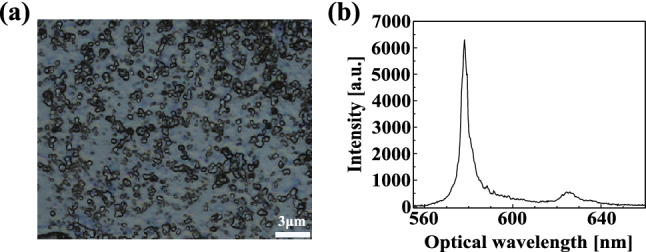
**(a)** An optical microscope image of hBN nanoflakes on a silicon substrate. **(b)** Example emission spectrum from defects in hBN.

We evaluate the size of hBN nanoflakes and the optical properties of defect centres in them. The defect centres are introduced into the nanoflake on silicon substrates by annealing at 850 $^{\circ }$C for 30 min under 1 Torr of argon atmosphere^28^. When the nanoflakes on a silicon substrate were observed using a 3D laser scanning confocal microscope (KEYENCE, VK-X1000), the size of them was roughly estimated to be 1 $\mu$m $\sim$ 2 $\mu$m, as shown in Fig.  1a.

Next, we evaluated the optical properties of defect centres in the nanoflakes on the substrate using a custom confocal microscope (See Method). The excitation power of the laser is 75 $\upmu$W. When we measured emission spectra at 22 bright spots in confocal microscope images, of which 17 spots showed two peaks: one is the ZPL with the full width at half maximum (FWHM) of about 10 nm at around 576 ± 6 nm and the other is a phonon sideband with FWHM of about 10 nm at around 621 ± 7 nm, as shown in Fig. 1b. These 17 spots were checked with Hanbury Brown-Twiss (HBT) and none were found to have single emission properties. This is due to the fact that the nanoflakes used in this work contained multiple defect centres in a range as small as the excitation laser spot (355 nm). Thus, it is considered that the defect centres in our samples on the substrate are a typical ensemble.

**Figure 2:**
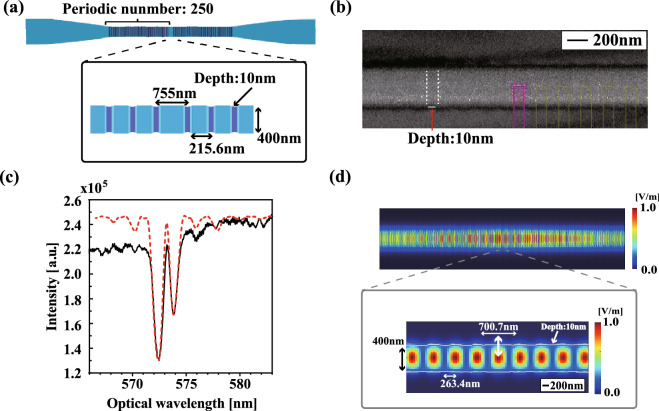
**(a)** The blueprint of fabricated NFBC. **(b)** SIM image of the fabricated NFBC. The dotted line shows the faint periodic grooves at $\sim$ 200 nm spacing on the fabricated nanofibre and the depth of about 10 nm. To clarify the image, the contrast of the image was adjusted. **(c)** Spectra of the fabricated NFBC (black line) and numerical curve (red dotted line). The resonant wavelength is 573 nm, and the linewidth is 0.71 nm. **(d)** Distribution of electric field intensity at the resonant wavelength of the NFBC.

### Optical properties and numerical analysis of the fabricated NFBCs

**Figure 3:**
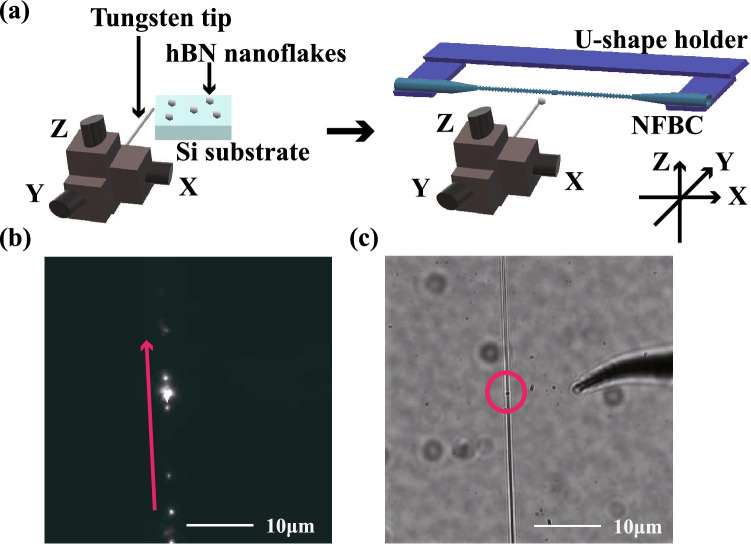
**(a)** Pick and place system for hBN nanoflakes. The left side illustrates the process for picking up an hBN nanoflake on a Si substrate by a tungsten tip on an XYZ stage. The right side illustrates placing the hBN nanoflake on an NFBC by the tungsten tip. The NFBC is fixed by a U-shape holder. **(b)** CCD image of the NFBC with a white light source. The bright spot is the centre of the NFBC. The red arrow indicates the direction of the nanofibre. **(c)** CCD image after placing the hBN nanoflake on the NFBC. The red circle indicates the hBN nanoflake on the NFBC. The tungsten tip can be observed on the right side of the nanofibre (black dots in the image show dirt on the surface of the CCD camera).

Based on the above results, we select the ZPL with a centre wavelength of 575 nm as the resonant wavelength of the NFBC. We fabricate an optical nanofibre with a waist diameter of $\sim$ 400 nm by heating a single-mode optical fibre (Thorlabs 630HP) with a ceramic heater and stretching it into a fine thread while maintaining single-mode propagation. The nanofibre is fixed on a U-shape metal holder, shown in Fig. 3a. A cavity structure on the nanofibre is fabricated using a He-ion FIB system with a fabrication resolution of less than 1 nm (Zeiss, Orion NanoFab)^33^. To realize a resonant wavelength close to the centre wavelength of 575 nm, the period of the grating and the length of the defect were set to 215.6 nm and 755 nm, respectively, in the FIB control system, as shown in Fig. 2a. The total number of period is also set to 250 on each side. A scanning ion microscope (SIM) image of the fabricated NFBC is shown in Fig. 2c. We observe faint periodic grooves with the depth of about 10 nm at $\sim$ 200 nm spacing on the fabricated nanofibre. The transmission spectrum (black color) of this fabricated NFBC is shown in Fig. 2c. The resonant wavelength is 573 nm, and the line width of this peak is 0.71 nm, with a Q-value of $\sim$ 807. The reduction of the transmission at the shorter wavelength side is due to the intensity dependence of the white light used as a light source.

To evaluate the fabricated NFBC, we numerically simulate the transmission spectrum, electric field intensity, and Purcell enhancement rate $\Gamma$ based on a three-dimensional (3D) finite-difference time-domain (FDTD) simulation (see methods). The numerically simulated spectrum (red dotted color) almost corresponds to the experimental spectrum as shown in Fig. 2c. The distribution of electric field intensity at this resonant wavelength is shown in Fig. 2d. The electric field intensity approaching the centre of the cavity increased exponentially with a period of 263.4 nm. We calculate $\Gamma$ at the resonant wavelength from $\Gamma = P_{\mathrm {cav}}/P_{\mathrm {0}}$, where $P_{\mathrm {cav}}$ is the power emitted by the dipole in the cavity and $P_{0}$ is the power radiated in the vacuum^12^. When a dipole with the radial orientation (a white double arrow in Fig. 2d) is placed on the surface of the cavity, $\Gamma$ is calculated to be 2.5. In this way, we succeeded in fabricating an NFBC with a resonant wavelength close to the centre wavelength of the ZPL of the defect centres in hBN.

### Transferring the hBN nanoflakes onto the NFBCs

We transfer a nanoflake to the fabricated NFBC using a sharp tungsten tip manipulated by a three-axis stage, as shown in Fig. 3a. First, the tungsten tip set on the stage is directed to the silicon substrate with the dispersed nanoflake. The nanoflake is picked up onto the tip and then placed at the centre point of the NFBC (bright spot in Fig. 3b) under observation using a CCD camera while propagating the light of the white light source in the NFBC. In this study, we tried to transfer hBN nanoflakes onto the NFBCs 21 times, of which 20 succeeded in transferring. However, the emission peak at the resonant wavelength of the NFBC was observed only once. As the result, the success rate was 4.8%. This would be due to the coupling of nanoflakes without defect centres, misalignment of the coupling position, and breaking of NFBCs during the experiment. Figure 3c shows a CCD camera image of the NFBC after transferring an hBN nanoflake onto it. The nanoflake is observed as a small round dot in the red circle. This position of the dot is the same as the bright spot in Fig. 3b. Note that the tungsten tip is also observed in the CCD camera image of Fig. 3c.

**Figure 4:**
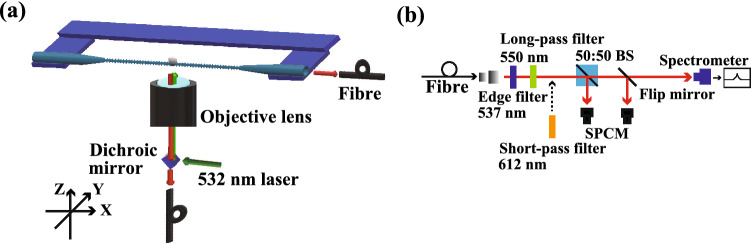
**(a)** Confocal microscope system for estimating an hBN nanoflake. A dichroic mirror separates the excitation light and the light emitted from the defect centres in hBN. **(b)** Detection system. A 537 nm edge filter and a 550 nm long-pass filter are placed before a 50:50 beam splitter (50:50 BS), single-photon counting modules (SPCMs), and a spectrometer. A 612 nm short-pass filter is inserted when the emission light is observed via the fibre end of the NFBC.

### Hybrid device of defect centres in hBN nanoflakes coupled to an NFBC

**Figure 5:**
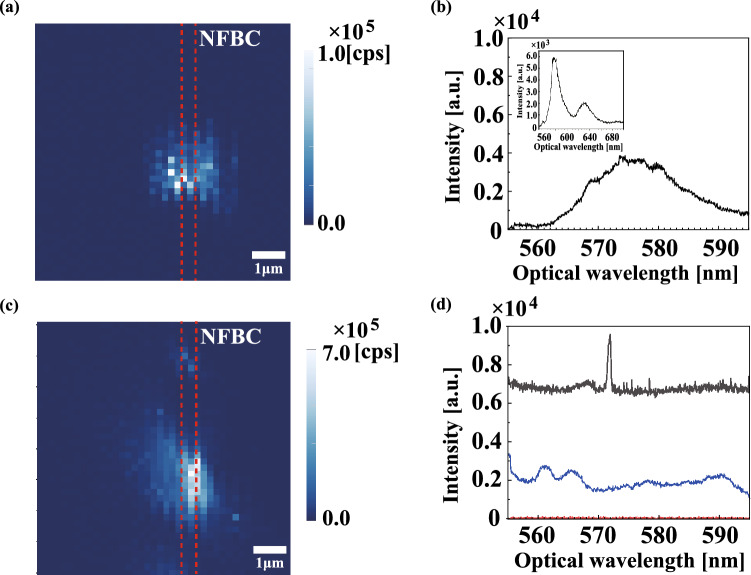
**(a)** Scanning image and **(b)** spectrum of the emission light from defect centres in an hBN on an NFBC via an objective lens. The region is around the centre wavelength of 575 nm on ZPL. The inset is the spectrum of all emission light from the defect centres. **(c)** Scanning image and **(d)** spectrum of the emission light from the defect centres in hBN via the fibre end (black curve). A sharp peak is observed around 572.6 nm. The blue curve shows the spectrum of background light without the nanoflake via the fibre end. The red line shows the spectrum of the emission light from only the NFBC via the fibre end, without the hBN nanoflake. These are excited by a pump laser through an objective lens. The red dotted line in **(a,c)** is the position of the NFBC.

We characterize the photoluminescence from defect centres in a nanoflake on an NFBC using a confocal microscope system (see Methods). The experimental setup is shown in Fig. 4a,b. The excitation power of the laser was 250 $\upmu$W. Figure 5a shows a confocal microscope image when the nanoflake is excited through the objective lens and the emitted photons are collected with the same objective lens. A bright spot is observed on the estimated position of the nanofibre (red dotted line). We measure the full emission spectrum of this bright spot, shown in the inset of Fig. 5b with the grooves of 300 groove/mm. Two emission peaks at $\lambda = 575$ nm and 630 nm are observed in the spectrum. The positions of the peaks almost agree with the typical wavelengths of the emission peaks from the nanoflake on the silicon substrate, as shown in Fig. 1b. However, the linewidth of the peak is broader than the example in Fig. 1b, likely due to the number of hBN layers^18^ and the size and shape of each nanoflake. To estimate the number of defect centres, we measure the second-order correlation function g$^2$($\tau$). $g^{(2)}(0)$ is almost unity, which means that the number of defect centres is much higher than one. Note that the degradation of the fluorescence was not observed when the nanoflake on an NFBC was excited for 2 h. In this way, we can confirm that an hBN nanoflake with defect centres is transferred to the NFBC from the Si substrate.

To confirm the coupling of the defect centres in the hBN nanoflake to the NFBC, we characterize the fluorescence through one end of the NFBC. Figure 5c shows a scanning image. A bright spot is observed at almost the same position as the nanoflake when the fluorescence is collected using the objective lens, shown in Fig. 5a. A black line in Fig. 5d shows an emission spectrum measured with the grating of 1800 groove/mm, when the bright spot is excited and the fluorescence is measured via the NFBC. Compared with the spectrum in Fig. 5b, a sharp emission peak at a wavelength of 572.6 nm is observed over the background light with almost constant intensity. This peak wavelength almost agrees with the resonant wavelength of the NFBC of 573 nm (Fig. 2c). To investigate the difference of 0.4 nm in wavelength, we measured the temperature dependence of the resonance wavelength while changing the temperature from 23 degrees to 28 degrees and found that the temperature shift was 0.67 nm / $^{\circ }$C. From this result, the difference of 0.4 nm would be due to the temperature change of about 0.6 $^{\circ }$C during the experiment. In this way, we have realized a hybrid device of defect centres in hBN nanoflakes and an NFBC.

Finally, to clarify the source of the emission peak at the resonant wavelength and the background light with almost constant intensity in the emission spectrum of the black curve in Fig. 5d, we investigate an NFBC without an hBN nanoflake and an NFBC with a defect-free hBN nanoflake. The red line in Fig. 5d shows the emission spectrum measured with the grating of 1800 groove/mm when the NFBC without an hBN nanoflake is excited with an excitation power of 150 $\upmu$W and the fluorescence is collected via the NFBC. The intensity of the spectrum is almost zero in the range of the observed wavelength. That is, we observe no fluorescence when only the NFBC was excited. Next, we investigate the NFBC with a defect-free hBN nanoflake. Note that this NFBC has a resonant wavelength of 577 nm and FWHM of 0.7 nm. The blue curve in Fig. 5d shows the emission spectrum when the fluorescence is measured via one end of the NFBC. The grating used in the spectrometer was 300 groove/mm. No emission peak is observed at the resonant wavelength of the NFBC, while a broad emission spectrum with almost constant intensity is observed. From these results, we consider that the emission from the defect centres was enhanced by the NFBC, while the broad background emission is due to the fluorescence of the impurities and defects existing in the fibre, excited by the pump light coupled to the nanofibre via nanoflakes as a scatterer. It is noted that the difference in the intensity between the blue curve and the background level of the black curve would be due to the difference in the coupling efficiency of the pump light to the nanofibre caused by the shape and size of the nanoflake.

## Conclusion

In conclusion, we have experimentally demonstrated a hybrid device of an NFBC and an hBN nanoflake with ensemble defect centres. We also observed a sharp emission peak at the resonant wavelength of 573 nm for the NFBC, close to the centre wavelength of 575 nm for the ZPL of defect centres in an hBN nanoflake. Our results open up a new direction of coupling photons emitted from stable quantum emitters with the narrow ZPL wavelength in defect centres into a single mode fibre, toward the realization of a fibre-integrated quantum information platform.

## Methods

### Numerical simulation for the fabricated NFBC

We numerically simulate the transmission spectrum and electric field intensity based on a three-dimensional (3D) finite-difference time-domain (FDTD) simulation^12^ using a commercial package (FDTD Solutions, Lumerical). The calculation area for the FDTD simulation is set to 120 $\times$ 2 $\times$ 2 $\upmu$m$^3$. The diameter of the nanofibre and the depth of the groove used in the calculation model are set to 400 nm and 10 nm, respectively. In the FDTD simulation, the depth of the groove is assumed to be 10 nm, which was obtained from the image analysis of the SIM image in Fig. 2b. The grating period of 263.4 nm, the defect length of 700.7 nm, and the refractive index (the nanofibre implanted with He ions) of 1.4555 were determined from the fit to the experimental transmission spectrum. The refractive index of the nanofibre without He ions is set to 1.45855 with the literature value of SiO$_2$^34,35^.

### Experimental setup

The experimental setup is shown in Fig. 3a. The confocal microscope system is composed of an IX 71 (Olympus) with a high NA objective (MPLAPON 100$\times$ / NA:0.95 Olympus) and a CCD camera (PRO EM 512 B, Princeton instruments). We use a 532 nm laser (Oxxius, LBX-532). The laser spot size is also 355 nm. The light from the laser is reflected by a dichroic mirror onto the nanoflake via an objective lens for optical excitation. The fluorescence from the nanoflakes is collected through the objective lens and the NFBC. After passing through the dichroic mirror, the fluorescence is collected with another objective lens, simplified in the diagram, and coupled to a multimode fibre with a core diameter of about 10 $\mu$m toward the detection system in Fig. 3b. Also, the fluorescence passing through the NFBC goes directly to the detection system. The detection system consists of a 537 nm edge filter and a 550 nm long-pass filter to cut the excitation light. We also use a 50:50 beam splitter (50:50 BS), single photon counting modules (SPCM-AQRH-14-FC, Excelitas Technologies) to observe the scanning image and the second-order autocorrelation function, $g^{(2)}(\tau )$, and a spectrometer (MS257, ORIEL Instruments) with a CCD camera (DU420-OE, Andor).
